# A Double-Layer Parallel MEMS Inductor with Enhanced Current-Carrying Capacity and Thermal Stability

**DOI:** 10.3390/mi17050571

**Published:** 2026-05-04

**Authors:** Xingyu Pi, Jiao Li, Hongyu Chen, Chunming Ren, Zhuoqing Yang, Chong Lei, Aiying Guo, Xuecheng Sun

**Affiliations:** 1 School of Mechatronic Engineering and Automation, Shanghai University, Shanghai 200444, China; xingyup@shu.edu.cn (X.P.); lijiaoshu@shu.edu.cn (J.L.); rcm@shu.edu.cn (C.R.); 2 Department of Micro-Nano Electronics, School of Electronic Information and Electrical Engineering, Shanghai Jiao Tong University, Shanghai 200240, China; yzhuoqing@sjtu.edu.cn; 3 School of Microelectronics, Shanghai University, Shanghai 200444, China

**Keywords:** double-layer inductor, microelectromechanical system (MEMS), current-carrying capacity

## Abstract

As a core component in electronic circuits, the size of inductors is crucial for the thin-film integration and miniaturization of circuits. Although various miniaturized inductors have been fabricated by using integrated circuit technology, their low current-carrying capacity and small inductance values cannot meet current application requirements. Therefore, this paper designs an inductor chip based on a double-layer parallel (DLP) array microcoil structure. Experimental verification demonstrates that the developed DLP inductor exhibits a far superior rated energy storage capability per unit area compared to other single-layer inductors, along with excellent thermal performance. Meanwhile, the 4 × 3 DLP array can withstand a maximum DC current of 4.25 A. This structural innovation provides a meaningful thermal–electromagnetic co-design reference solution for highly reliable integrated power modules.

## 1. Introduction

Driven by the exponential growth in High-Performance Computing (HPC) and Artificial Intelligence (AI) accelerators, the demand for Fully Integrated Voltage Regulators (FIVRs) and Power-Supply-on-Chip (PwrSoC) architectures has reached unprecedented levels [1,2]. To achieve ultimate system miniaturization and high-frequency power delivery, these advanced power management systems heavily rely on embedded passive components. Within this framework, on-chip micro-inductors have emerged as the primary bottleneck, as they strictly require massive current-handling capabilities and thermal performance while confined to an ultra-compact footprint [3].

To address the stringent requirements of integration compatibility, MEMS thin-film inductors provide an effective solution by virtue of their compact structure and CMOS-compatible fabrication processes, thus becoming a prominent research focus in the monolithic integration of power management systems at present [4,5,6,7]. However, the application of existing MEMS thin-film inductors under high-power operating conditions is still plagued by two core bottlenecks: insufficient current-handling capability and severe thermal performance degradation [8,9,10]. As operational current densities continually escalate, the resulting excessive Joule heating poses a severe threat to both device reliability and overall system efficiency.

To tackle these thermal and current-carrying limitations, multilayer/dual-layer structural innovations have emerged as a key research direction, leveraging the advantages of stacked conductors to enhance both electrical and thermal performance. Historically, the concept of utilizing multilayer or two-layer structures in micro-inductors has been explored to address various performance limitations. As early as 1998, Yue and Wong proposed on-chip spiral inductors with patterned ground shields for silicon-based RF ICs to improve the quality factor [11]. Subsequent studies by Ragonese et al. established scalable physics-based models for silicon spiral inductors, paving the way for multilayer designs [12]. Furthermore, innovative multilayer configurations have been patented and developed to achieve unique electrical properties, such as negative inductance structures proposed by Widjaja and Sarangan [13], and high-performance microcoils using layered films [14].

However, despite these advancements in two-layer and multilayer geometries, most existing designs primarily focus on maximizing spatial efficiency, optimizing radio-frequency (RF) characteristics, or achieving specific electromagnetic shielding on traditional silicon substrates. When applied to modern high-power operating conditions (e.g., currents exceeding 2–3 A), these conventional geometries are still trapped in a fundamental physical trade-off: the “electrothermal deadlock”. Expanding the conductor volume to enhance the current rating inevitably consumes valuable chip area. Furthermore, traditional silicon substrates suffer from severe substrate eddy current losses, which degrade high-frequency signal integrity [15,16,17].

To overcome these structural and material bottlenecks, this paper proposes an inductor chip based on a DLP array microcoil structure, fabricated via advanced MEMS processes. Our approach introduces three distinct advantages over prior methods: First, unlike traditional series-stacked structures, our unique parallel array architecture evenly distributes the input current to the upper and lower coils, directly halving the localized current density and substantially suppressing Joule heating while maintaining high inductance through vertical magnetic coupling. Second, by utilizing MEMS technology, the device achieves an ultra-thin and compact profile, making it highly compatible with the stringent dimensional requirements of modern thin-film integration and emerging flexible electronics. Finally, substituting the conventional silicon wafer with a glass substrate eliminates substrate eddy currents, ensuring superior electrical insulation and maintaining excellent signal integrity even under high-frequency and high-power operations. This structural and material innovation sets a new benchmark for thermal-electromagnetic co-design, making its rapid dissemination highly impactful for the immediate advancement of ultra-high-density PwrSoC modules.

## 2. Double-Layer Inductor Model Design

**Figure 1:**
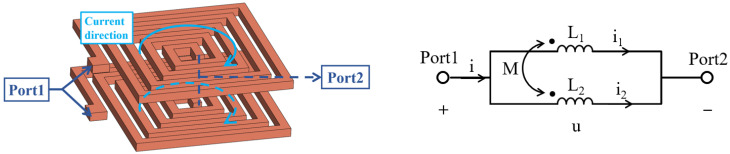
Double-layer parallel coils and the equivalent circuit.

Based on the single-layer coil, we extended it to a double-layer parallel structure, as illustrated in Figure 1. According to Kirchhoff’s current law, the total inductance is given by:

(1) $$L_{\mathrm{total}}=\frac{L_{1}L_{2}-M^{2}}{L_{1}+L_{2}-2M}$$

Since ideal coupling does not exist in practical scenarios, the total inductance cannot be zero. In general, the double-layer parallel coil exhibits a total inductance comparable to that of a planar parallel coil.

**Figure 2:**
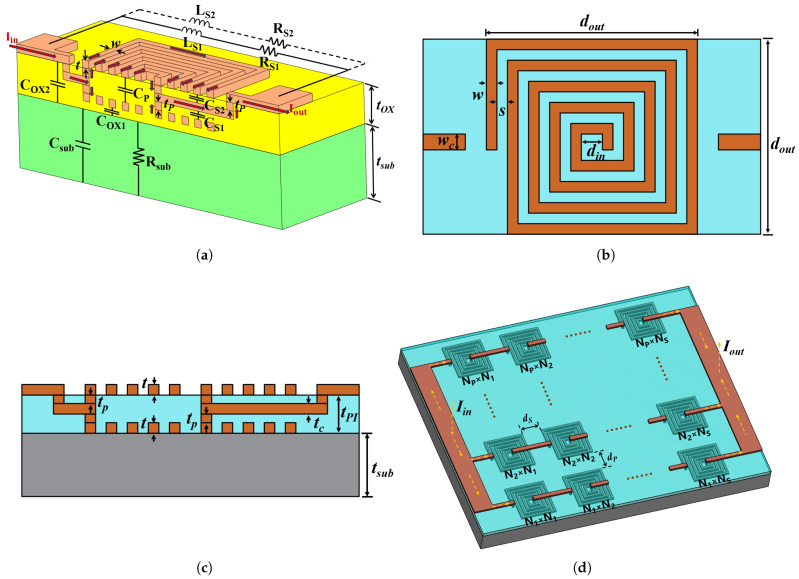
(**a**) 3-D view Equivalent circuit model of the MEMS double-layer thin-film inductor with parallel connection (Red arrows represent the current path). (**b**) Top view. (**c**) Cross view. (**d**) 3-D view of the array inductor.

The geometric model of the DLP thin-film inductor features a five-layer configuration (Figure 2a–c). During operation, current enters via the top pad, passes through the intermediate routing layer, splits equally into the two coil layers, recombines at the opposite routing layer, and exits through the other top pad. In Figure 2a, the physical meanings of $C_{\mathrm{sub}}$, $R_{\mathrm{sub}}$, $C_{OX1}$, $C_{OX2}$, $L_{1}$, $R_{S1}$, $L_{2}$, and $R_{S2}$ are consistent with those in the equivalent circuit model of the double-layer series thin-film inductor. The magnetic flux superposition of the two coils is represented by the mutual inductance in the same-name terminal parallel configuration. Among these parameters, the calculation formula for $C_{OX1}$ is modified as follows:

(2) $$C_{OX1}=\frac{1}{2}lw\frac{\varepsilon_{OX}}{t_{OX}-2\left(t_{p}+t\right)}$$

Furthermore, $C_{S1}$ and $C_{S2}$ denote the overlap capacitances between the bottom/top coils and the intermediate connecting lines. Since the structures of the top and bottom coils are identical, these two capacitances are equal. Due to the structural modification, the overlap capacitance $C_{P}$ between the coils is altered. Specifically, the calculation formulas for $C_{S1}$, $C_{S2}$, and $C_{P}$ are:

(3) $$C_{S1}=C_{S2}=\frac{\varepsilon_{OX}}{t_{p}}nw^{2}$$

(4) $$C_{P}=\frac{\varepsilon_{OX}}{2t_{p}+t}lw$$

To illustrate this theoretical background with practical values, We consider the base geometric parameters of our designed DLP inductor (detailed later in Section 4, Table 1). For an example structure with a conductor width w=50μm, wire thickness t=50μm, pillar thickness $t_{p}=40\;\mu m$, and utilizing a polyimide layer with a relative permittivity ($\varepsilon_{r}\approx 3.4$), the parasitic capacitances ($C_{S1}$, $C_{S2}$, and $C_{P}$) are effectively restricted to the sub-picofarad (pF) range. Furthermore, by plugging in these physical dimensions and the layout footprints, the baseline single-cell double-layer parallel structure is estimated to achieve a total theoretical inductance ($L_{total}$) of approximately 12.76nH. These practical baseline values demonstrate that the rationally designed geometric parameters not only safely control the parasitic elements, thereby guaranteeing a high self-resonant frequency (SRF) for the double-layer parallel configuration, but also halve the branch currents to mitigate Joule heating.

## 3. Simulation of Double-Layer Inductor

It can be observed that $C_{S1}$, $C_{S2}$, and $C_{P}$ are all positively correlated with the coil conductor width and negatively correlated with the thickness $t_{P}$ of the vertical interconnects. Reducing the conductor linewidth or increasing the thickness of the vertical interconnects can effectively decrease the above capacitance values.

This parallel current division halves the load per coil layer, significantly mitigating Joule heating and boosting the maximum current-carrying capacity. Although the halved current reduces the individual magnetic flux of each layer, the vertical superposition of the total flux effectively compensates for this reduction. Consequently, the inductance of the DLP structure experiences only negligible degradation. This basic structure is laterally scaled into an $N_{p}$ × $N_{s}$ array configuration (where *p* and *s* denote rows and columns) by horizontally interconnecting multiple individual inductors (shown in Figure 2d).

In this study, the finite element method (FEM) was employed to simulate and analyze MEMS thin-film inductors using the commercial simulation software COMSOL Multiphysics 6.0. The objective was to investigate the influence of the geometric structure of MEMS thin-film inductors on their electrical and thermal performance, thereby optimizing the detailed parameters for the structural design of MEMS thin-film inductors. Based on our previous research on single-layer inductors, we simulated the performance of double-layer series and double-layer parallel inductors, with a fixed vertical interconnect thickness of $t_{c}=50\;\mu m$.

**Figure 3:**
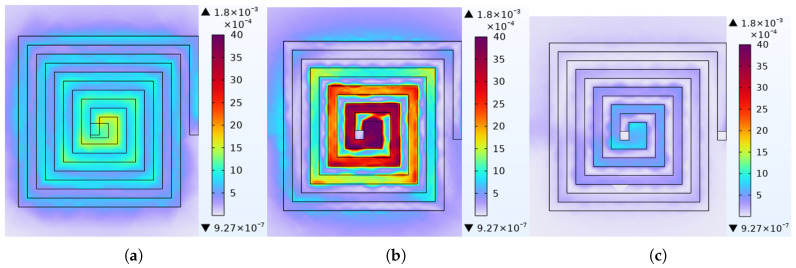
Magnetic flux density distributionof MEMS thin-film inductors: (**a**) Single layer. (**b**) Double layer (series). (**c**) Double layer (parallel).

Figure 3 presents the finite element simulation results of the magnetic flux density distribution and magnetic flux streamline distribution for the three types of MEMS thin-film inductors. The upper and lower limits of the color bar are set identically to facilitate comparative analysis. Furthermore, all three inductor structures were excited by an alternating current (AC) with a frequency of 1 GHz and a magnitude of 0.1 A. Evidently, the double-layer series structure exhibits the best inductive performance, while the double-layer parallel structure shows the weakest performance, although the difference from the single-layer structure is relatively small.

**Figure 4:**
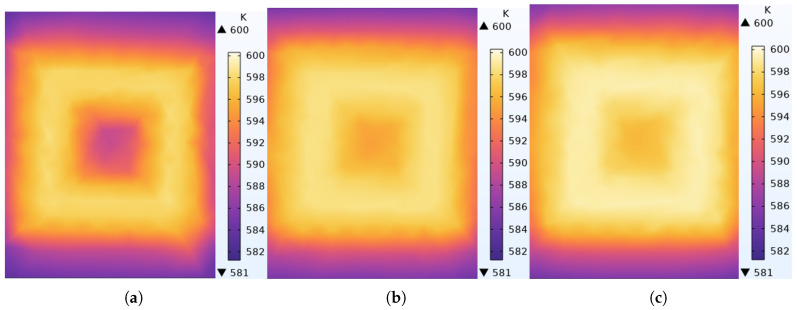
Temperature distribution of polyimide layers in MEMS thin-film inductors with 3 different structures: (**a**) Single layer. (**b**) Double layer (series). (**c**) Double layer (parallel).

Figure 4 illustrates the finite element simulation results of the temperature distribution in the PI layer for the three MEMS thin-film inductor structures. All inductors were excited by a direct current (DC), and the maximum temperature of the PI layer was controlled at approximately 600 K. The temperature contour maps reveal that the most intense heat generation occurs around the middle-turn conductors rather than at the center of the coil, and the temperature distributions of the three thin-film inductors are generally consistent. Meanwhile, the double-layer parallel structure demonstrates slightly better thermal performance than the other two structures.

**Figure 5:**
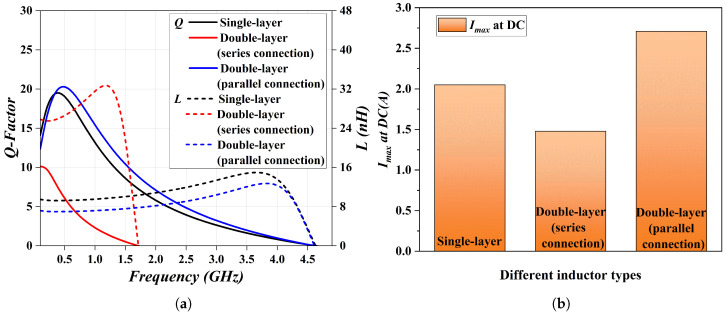
Performance comparison of MEMS thin-film inductors with 3 different structures: (**a**) Electrical performance. (**b**) Maximum current-carrying capacity.

Figure 5 quantitatively analyzes other performance metrics of the three structures. The simulation results indicate that the electrical performance of the double-layer parallel thin-film inductor is close to that of the single-layer inductor. It exhibits a slightly higher maximum quality factor (19.644@0.4 GHz for the single layer vs. 20.39@0.5 GHz for the double-layer parallel) and a slightly lower maximum inductance (14.974 nH@3.7 GHz for the single layer vs. 12.76 nH@3.8 GHz for the double-layer parallel), along with superior frequency characteristics. In addition, the double-layer parallel thin-film inductor achieves the optimal maximum current-carrying capacity of 2.71 A, representing improvements of 32.2% and 83.2% compared with the single-layer and double-layer series structures, respectively. In contrast, the double-layer series structure outperforms the other two structures only in terms of inductance value.

**Figure 6:**
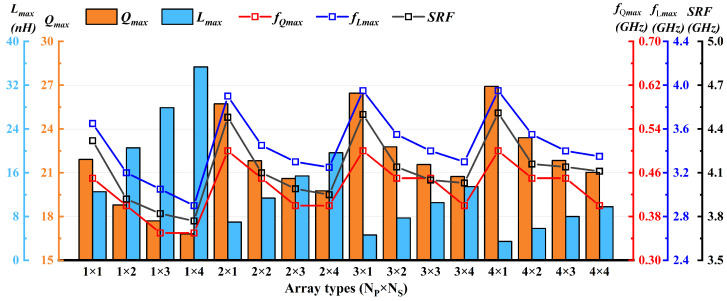
Electrical performance of double-layer (parallel connection) array inductors.

To further investigate the effect of arrayed coils on inductor performance, this study quantitatively analyzed the electrical performance of various arrays for the three inductor structures, with the simulation results shown in Figure 6. Taking the single-cell array ($N_{s}$ = 1, i.e., 1 × 1/2 × 1/3 × 1/4 × 1) as the benchmark, each additional stage of planar series connection increases $L_{\max}$ by an average of 76.4%, 135.29%, and 194.11%, respectively, while $Q_{\max}$, $f_{Q_{\max}}$, and $f_{L_{\max}}$ decrease slightly. Taking the single-row array ($N_{p}$ = 1, i.e., 1 × 1/1 × 2/1 × 3/1 × 4) as the benchmark, each additional stage of planar parallel connection reduces $L_{\max}$ by an average of 48.73%, 59.66%, and 75.89%, respectively, whereas $Q_{\max}$ increases by an average of 15.90%, 19.09%, and 22.7%, respectively. Moreover, increasing the number of series or parallel stages results in a slight reduction in the self-resonant frequency (SRF).

**Figure 7:**
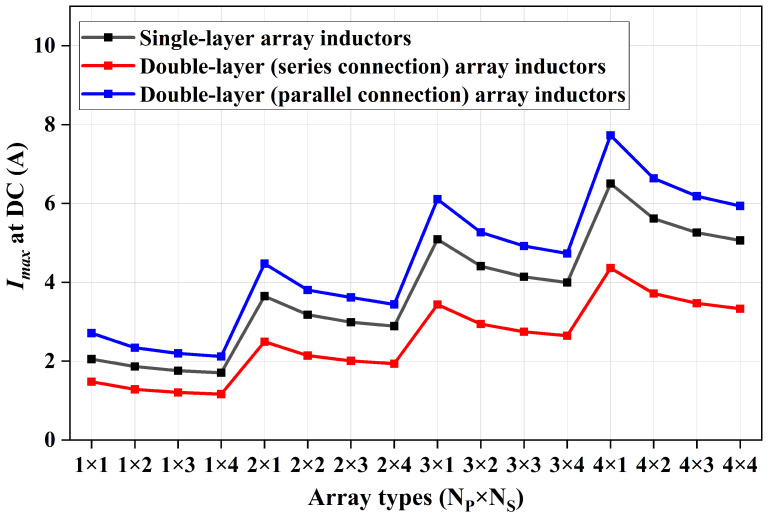
Maximum current-carrying capacity of array inductor.

Similarly, the maximum current-carrying capacity of various arrays for the three inductor structures was quantitatively analyzed, with the simulation results presented in Figure 7. The results show that, relative to the single-cell array ($N_{s}$ = 1), each additional stage of planar series connection reduces the maximum current-carrying capacity by an average of 13.48%, 18.83%, and 21.85%, respectively. In contrast, relative to the single-row array ($N_{p}$ = 1), each additional stage of planar parallel connection improves the maximum current-carrying capacity by an average of 67.44%, 130.56%, and 191.74%, respectively.

Based on the above quantitative analysis, the key conclusions can be drawn as follows: The double-layer parallel structure offers significant advantages in achieving higher current-carrying capacity and better thermal performance with only minor degradation in electrical performance. Furthermore, arraying the double-layer structures can further enhance their electrical and thermal performance.

## 4. Fabrication of Inductor

Based on the simulation results in the previous section, the structural parameters of the double-layer parallel inductor were determined in this study, as listed in Table 1.

**Table 1:** Geometric Parameters of Designed DLP Inductor.

Parameter	Items	Values (μm)
*t*	thickness of spiral wires	50
$t_{p}$	thickness of pillars	40
$t_{\mathrm{PI}}$	thickness of polyimide layer	90
$t_{c}$	thickness of connecting conductors	50
$t_{\mathrm{sub}}$	thickness of glass substrate	1800
*w*	width of spiral wires	50
$w_{c}$	width of connecting conductors	200
$w_{p}$	width of pillars	100
*s*	spacing of the lines of spiral wires	50
$d_{s}$	x-axis spacing of arrayed coils	500
$d_{p}$	y-axis spacing of arrayed coils	500
$d_{\mathrm{in}}$	inner diameter of the coil	50
$d_{\mathrm{out}}$	outer diameter of the coil	1000

The fabrication of this thin-film inductor primarily utilizes MEMS processes such as photolithography, sputtering, etching, and electroplating, proceeding sequentially through the five-layer structure. All fabrication processes in this work were carried out on 4-inch glass wafers. To make full use of the limited wafer area while ensuring the quantity and quality of qualified samples, the number of prepared arrays of different configurations was not uniform. In addition, to guarantee reliable interconnection between conductors in different layers, the cross-sectional area of the vertical interconnects was slightly enlarged in the design, together with an increased contact area between the coils and their connecting sections. This structural variation has a negligible effect on the performance of the array inductors.

**Figure 8:**
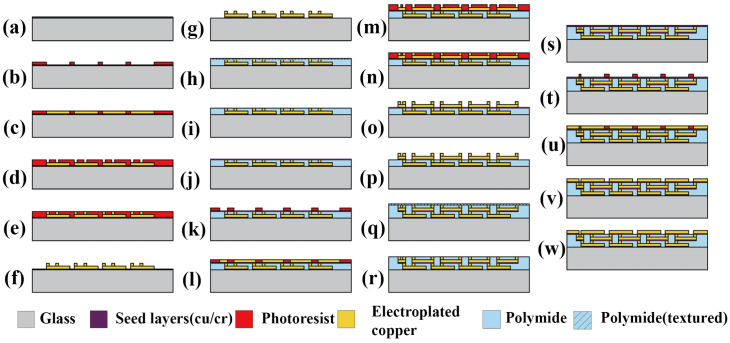
Fabrication process of double-layer array inductors with parallel connection ($N_{s}$ = 4).

The DLP array inductor, possessing a five-layer structure, requires a more complex fabrication process compared to single-layer or standard double-layer series inductors [18]. The fabrication process flow of the MEMS thin-film inductor is illustrated in Figure 8, with detailed steps as follows:

Figure 8a A Cr/Cu seed layer (10 nm Cr and 150 nm Cu) was deposited on the glass substrate.

Figure 8b Positive photoresist with a thickness of approximately 50 μm was spin-coated on the seed layer. After baking the sample on a hot plate at 90 °C for 2 h, conventional photolithography was performed to pattern the photoresist and form the electroplating mold for the bottom-layer coil.

Figure 8c Copper was electroplated through the mold to form the bottom coil.

Figure 8d second layer of photoresist with a thickness of approximately 40 μm was spin-coated over the previous layer, followed by patterning to create the mold for vertical interconnects.

Figure 8e Copper was electroplated to form the vertical interconnects.

Figure 8f All photoresist was removed using acetone and ultrasonic agitation for 10–15 min.

Figure 8g The first seed layer was stripped via dry etching. The substrate was then cleaned and dried on a hot plate at 90 °C for 1 h.

Figure 8h Polyimide (PI) was spin-coated to fill the gap between the previous two layers, with the filling height slightly higher than the vertical interconnects to ensure full coverage. The sample was subsequently cured in a vacuum oven at 250 °C for 2 h.

Figure 8i The PI layer was finely polished until the surface of the vertical interconnects was exposed, yielding a flat processing plane.

Figure 8j A Cr/Cu seed layer with the same thickness as above was deposited.

Figure 8k Positive photoresist with a thickness of approximately 50 μm was spin-coated on the seed layer. Following the same baking and patterning procedures, the electroplating mold for the top-layer interconnects (or coils in the double-layer series configuration) and lead pads was fabricated.

Figure 8l Copper was electroplated through the mold.

Figure 8m Another layer of photoresist with a thickness of approximately 40 μm was spin-coated and patterned to form the mold for a second set of vertical interconnects.

Figure 8n Copper was electroplated to form the second vertical interconnects.

Figure 8o All photoresist was removed using acetone and ultrasonic agitation for 10–15 min.

Figure 8p The second seed layer was removed by dry etching. The substrate was cleaned and dried at 90 °C for 1 h.

Figure 8q PI was spin-coated to fill the gap between the layers, with a height slightly above the vertical interconnects to ensure complete filling, followed by curing in a vacuum oven at 250 °C for 2 h.

Figure 8r The PI layer was polished until the vertical interconnects were exposed, achieving a flat surface.

Figure 8s A Cr/Cu seed layer (10 nm Cr, 150 nm Cu) was deposited.

Figure 8t Photoresist with a thickness of approximately 50 μm was spin-coated and patterned using the same baking and lithography procedures to form the mold for top-layer interconnects and lead pads.

Figure 8u Copper was electroplated through the mold.

Figure 8v All photoresist was stripped using acetone and ultrasonic treatment for 10–15 min.

Figure 8w The top seed layer was removed via dry etching. The substrate was cleaned and dried at 90 °C for 1 h, completing the fabrication process of the double-layer parallel array inductor.

**Figure 9:**
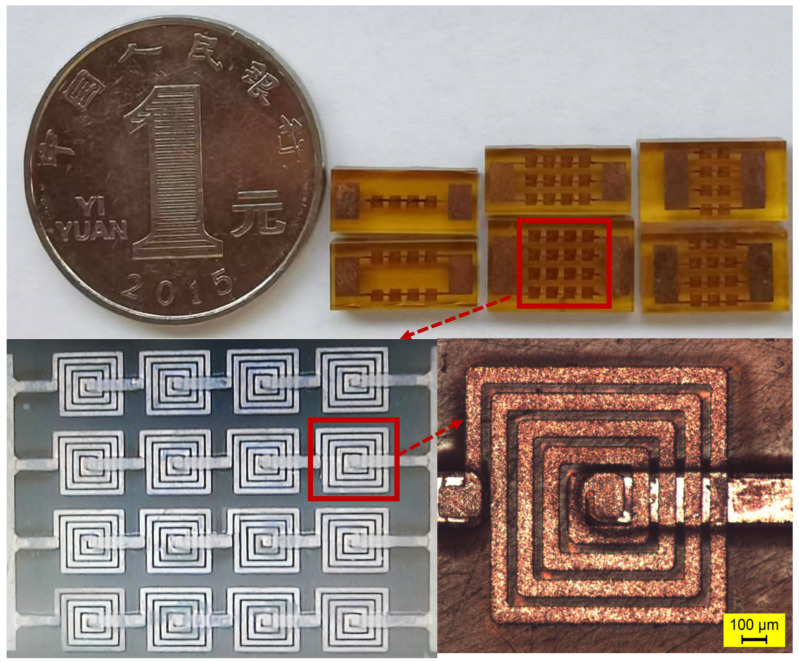
Fabricated double-layer parallel array inductors.

In this study, the double-layer parallel inductors were successfully fabricated and taped out, with all fabricated chips using 4-inch glass wafers as substrates. Figure 9 presents partial tape-out results of the double-layer array inductors, and all tests were performed on the successfully fabricated samples.

## 5. Test Methods and Results Analysis

### 5.1. Electrical and Thermal Performance Test

Electrical performance was measured using an impedance analyzer, which operates on the auto-balancing bridge method, covering a frequency range from 100 Hz to 40 MHz. The actual *L* and quality factor (*Q*) of the thin-film inductor samples were calculated using the following formulas:

(5) $$L_{c}=L_{s}-L_{w}$$

(6) $$Q_{c}=\frac{X_{s}-X_{w}}{R_{s}-R_{w}}$$

where $L_{s}$, $X_{s}$, and $R_{s}$ are the measured inductance, reactance, and resistance of the device under test including the connecting leads; $L_{w}$, $X_{w}$, and $R_{w}$ are the measured inductance, reactance, and resistance of an identical length of the connecting leads alone [19].

To simulate the inductor’s behavior under extreme operating conditions, it is essential to accurately measure the critical current that leads to device failure. By gradually increasing the applied current, the point at which the inductor’s resistance exhibits a significant, order-of-magnitude increase is recorded; this value is defined as the maximum current ($I_{\max}$). Concurrently, this study adopts a conservative temperature rise ΔT of 25 °C as the safety design constraint. For coreless inductors, thermal dissipation is exclusively dominated by the ohmic losses of the windings. To suppress the positive feedback effect induced by the (ΔT) increase in high-frequency AC resistance and to prevent severe degradation of the *Q*, limiting the temperature rise to 25 °C effectively restricts the thermally induced resistance drift of the copper wire to a reasonable margin of within 10% [20,21]. The current measured at this thermal threshold is designated as the rated current ($I_{r}$).

To evaluate the comprehensive parameters of inductors and quantify the temperature performance and inductance utilization of different planar micro-inductors, we introduce the figure of merit (FOM) for inductance as a comprehensive evaluation index. The calculation formula expressed as

(7) $$FOM=\frac{L_{c}\cdot I_{r}^{2}}{s}$$

where *s* is the effective area of the inductor. In general, the term $\frac{1}{2}L\cdot I^{2}$ represents the energy storage capability of an inductor, and thus this index characterizes the energy storage capability per unit area under $I_{r}$ [3,22]. A higher value of this index indicates a greater energy storage capability per unit area and a stronger current-handling capability at 25 °C temperature rise, enabling an intuitive comparison of the comprehensive performance of coreless planar micro-inductors with different dimensions and structures.

### 5.2. Results Analysis

**Figure 10:**
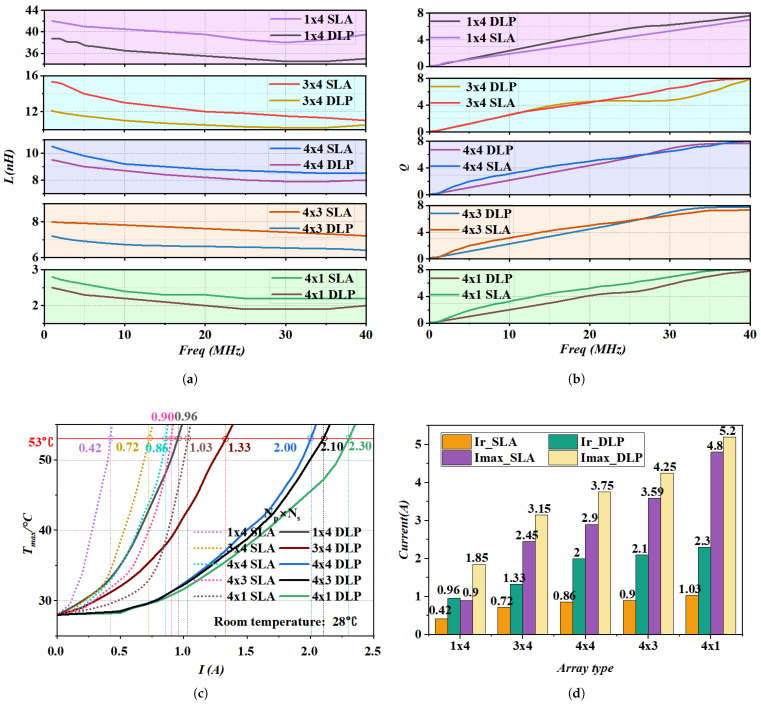
(**a**,**b**) Electrical performance test results of single and double array inductor, (**c**) Current-Temperature curves for various array inductors. (**d**) Maximum current and rated current of single-layer and double-layer inductors under different array configurations.

Following the method described above, we selected high-quality double-layer array inductors for electrical and thermal performance characterization. Figure 10a,b show the measured *L* and *Q* versus frequency for the array configurations, which indicate that the maximum inductance ($L_{\max}$) loss rates for the 1 × 4, 3 × 4, 4 × 4, 4 × 3, and 4 × 1 arrays were 7.8%, 22.5%, 9.5%, 10% and 10.7% compared to the SLA structure, respectively. Furthermore, the *Q* for both structures remained comparable across the entire measured frequency range. Notably, the 4 × 4 DLP array achieves a $L_{\max}$ close to 10 nH, with $Q_{\max}$ reaching 7.7.

Figure 10c compares the steady-state temperatures of SLA and DLP arrays inductor under various DC loads, demonstrating the latter’s superior thermal management. Consequently, the rated currents across the 1 × 4, 3 × 4, 4 × 4, 4 × 3, and 4 × 1 DLP arrays improved by 128%, 84%, 132%, 133%, and 123%, respectively. Furthermore, Figure 10d highlights significant gains in $I_{\max}$. Notably, the 4 × 3 DLP sustained up to 4.25 A—a 22% increase over the SLA’s 3.59 A—with overall $I_{\max}$ improvements of 105%, 28.5%, 29.3%, 18.3%, and 8.3% across the aforementioned geometries.Evidently, under the same array configuration, the double-layer parallel structure can significantly improve the current-carrying capacity with only a slight reduction in inductance. Moreover, the FOM of the DLP inductor is more than four times that of the SLA inductor, demonstrating excellent thermal performance under rated operating conditions.

Meanwhile, we compared the comprehensive performance of different inductors, as presented in Table 2. The $I_{\max}$ and FOM of our 4 × 4 DLP inductor are significantly superior to those of the other inductors. Reference [23] made every effort to etch trenches in the silicon substrate and fill them with thick copper to enhance the current-carrying capacity. However, limited by the single-layer structure, the rated current was stuck at only 0.6 A, the maximum current was merely 2 A, and the FOM was only 5.89, which is much lower than that of our DLP inductor. Reference [24] adopted the complex through-silicon via (TSV) technique combined with suspended coils. The inductor area reached 9 mm^2^, and although the inductance was considerably improved, its rated current was only 1 A, with an FOM of less than 5. As discussed previously, the DLP array inductor exhibits excellent current and temperature performance with a compact footprint.

**Figure 11:**
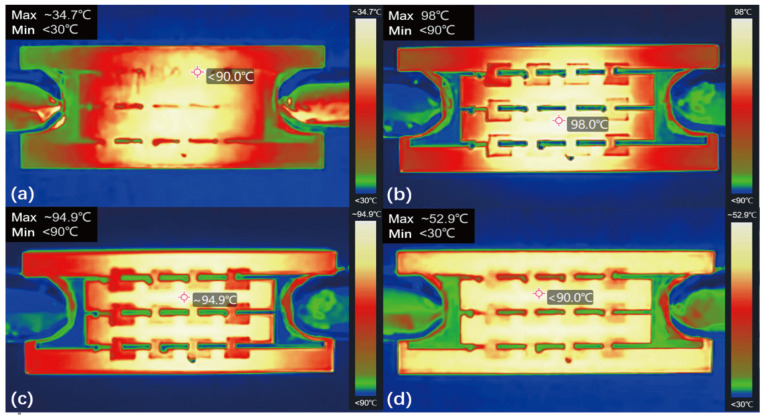
Temperature comparison of the 3 × 4 DLP inductor at 2.2 A DC input for (**a**) t < 5 s, (**b**) steady state. Disconnection of 2.2 A DC input for (**c**) t < 5 s, (**d**) t > 10 s.

Figure 11 illustrates the transient heating and cooling stages of the 3 × 4 DLP array inductor under a 2 A load. During the heating process, localized hot spots initially emerge at the array’s center and subsequently diffuse across the entire planar surface, with the peak steady-state temperature concentrated near the central coils. Conversely, during the cooling process after current disconnection, the device rapidly dissipates heat through the polyimide layer. As the temperature drops, the thermal outlines of the central coils briefly sharpen before the entire chip thermally equilibrates. Overall, these thermal analyses confirm that the DLP array exhibits superior thermal performance and robust high-current stability compared to the SL inductor.

## 6. Conclusions

In this work, to develop micro-inductor devices with higher current-carrying capacity and better thermal performance, modeling and simulation were carried out for single-layer, double-layer series, and double-layer parallel array structures, which were further extended to various array configurations. Based on theoretical and simulation results, double-layer parallel inductors with 1 × 4, 3 × 4, 4 × 4, 4 × 3, and 4 × 1 arrays were designed and successfully fabricated. The measured results demonstrate that, compared with conventional single-layer and double-layer series structures, the proposed double-layer parallel inductors exhibit significantly enhanced current-carrying capacity and greatly improved temperature rise performance. In particular, the 4 × 3 array DLP inductor achieves a maximum current of 4.25 A and a rated current of 3.59 A. This provides a promising solution for high-current, compact application scenarios, such as PwrSoC.

Nevertheless, the performance of our devices can be further improved. First, the fabrication process can be optimized. Owing to the complexity of the multilayer process, the yield of the taped-out chips is relatively low. In future work, we will gradually optimize the process at each stage according to failure analysis to reduce the defect rate. Second, the inductance reduction caused by the parallel structure can be minimized. By adjusting the spacing between the double-layer coils, the mutual inductance can be effectively enhanced, thereby increasing the overall inductance and reducing the inductance loss. Furthermore, manipulating the heat distribution and current flow regime offers exciting avenues for future exploration. As current density scales up, the distribution of electric and magnetic fields within the inductor becomes highly complex. Future designs could explore intentionally creating transverse magnetic field gradients—potentially by integrating artificial magnetic materials (e.g., neodymium magnets) between layers. Utilizing the resulting Lorentz force to alter the current distribution across the conductors could introduce a novel mechanism to actively control current limits and further optimize thermal fields in next-generation micro-inductors.

## Figures and Tables

**Table 2:** Comparison of Different Inductors.

Reference	Structure	$I_{\max}$/A	$L_{\max}$/nH	$I_{r}$/A	$s/{\mathrm{mm}}^{2}$	FOM
This work	4 × 4 DLP	3.75	10.46	2	1	41.84
[17]	4 × 4 SLA	2.95	13.26	0.86	1	9.81
[23]	Embedded SL (Coreless)	2	13.1	0.6	0.8	5.90
[24]	3D Toroidal (Air-core)	2 (Simulated)	44.6	1	9	4.95

## Data Availability

Dataset available on request from the authors.
